# Vesicourethral Reflux in Pediatrics With Hypermobility Syndrome

**DOI:** 10.5812/numonthly.10770

**Published:** 2013-08-12

**Authors:** Fatemeh Beiraghdar, Zohreh Rostami, Yunes Panahi, Behzad Einollahi, Mojtaba Teimoori

**Affiliations:** 1 Nephrology and Urology Research Center, Baqiyatallah University of Medical Sciences, Tehran, IR Iran

**Keywords:** Hypermobility syndrome, Pediatrics, Vesico-Ureteral Reflux, Urologic Diseases, Urinary Tract Infections

## Abstract

**Subjectives:**

Prevalence of benign joint hypermobility syndrome (BJHMS) without systemic disease seems to be high in children. Little literature is currently available related to urinary tract diseases in patients with BJHMS. Here, we report an association between the urinary tract disease and BJHMS.

**Methods:**

We conducted a prospective case series study of 62 pediatric patients with musculoskeletal pain to detect urinary tract diseases in Tehran, Iran from October 2009 to October 2010. The Brighton criteria score was used to diagnose BJHMS. The collected data included age, gender, grading of vesicoureteral reflux (VUR), ultrasonography findings, urodynamic results and biochemical tests. Voiding cystourethrography was used for detection and grading of VUR.

**Results:**

VUR was observed in 60% of patients with BJHMS. However, sonography was normal in 66.7% of patients. The most common grading of reflux was grade II of VUR (37.5%). Seventy percent of patients with BJHMS and neurogenic bladder had failure to thrive.

**Conclusion:**

Our findings showed an increased frequency of VUR in patients with BJHMS. We suggest that Infants and children with BJHMS should be screened for VUR.

## 1. Introduction

The musculoskeletal pain is a common problem in children, and it is important to distinguish its causes (1). This type of pain in children is commonly caused by benign joint hypermobility syndrome (BJHMS) and growing pains. BJHMS is an inherited connective tissue disorder with hypermobility of joints with no swelling and tenderness in the absence of a systemic rheumatologic disease (1, 2). The prevalence of BJHMS without systemic disease is 4% to 13% of the general population (3). In a study, BJHMS occurs in 66% of school children with arthralgia of unknown etiology (4). Disorder in different organs may result in a wide variety of clinical features, and disabilities can be seen in BJHMS (2, 5).

Renal diseases such as focal and segmental glomerulonephritis (6), polycystic kidney disease (7) and medullary sponge kidneys (8) may occur in these patients. Vesicoureteral reflux (VUR) is a common childhood problem, and may lead to the development of renal scarring with subsequent renal failure (9). The incidence is high, especially in patients diagnosed by urinary tract infection (UTI). Early detection and treatment may prevent further UTIs and chronic kidney disease. Thus, detection of association of VUR with other clinical conditions is very important. To our knowledge, there is no such concomitant with BJHMS in literature and there has been little data on association between urinary tract disease and BJHMS.

Only a few data are currently available in literature regarding the kidney and urological problems in patient with connective tissue disorder (10). Here, we report a relationship between urinary tract disease and BJHMS.

## 2. Patients and Methods

We conducted a prospective case-series study to investigate the relationship between BJHMS and urinary tract diseases in a pediatric clinic at Baqiyatallah medical school from October 2009 to October 2010. Sixty-two patients under 14 years old with musculoskeletal pain referred to us for urinary tract diseases examination. The Brighton criteria (11) were used to diagnose hypermobility syndrome, and BJHMS was also confirmed by a pediatric rheumatologist. Four out of 62 patients were lost to follow up.

We excluded the patients with other causes of joints pain from BJHMS by patient history, physical examinations and laboratory data such as complete blood cell count, erythrocyte sedimentation rate, anti-nuclear antibody, anti-double stranded DNA antibody, serum complements (C3, C4 and CH50), rheumatoid factor and antistreptolysin O titer.

Fifteen out of 58 patients (26%) had BJHMS. Subsequently, the age, sex, family history of renal diseases and BJHMS, urinary tract infection, history or current renal stone and rheumatologic disorders were recorded. In addition, history of urinary frequency, enuresis, incontinency, constipation, macroscopic hematuria and infrequent voiding were also recorded.

Urine culture, liver function test, cell blood count, blood urea nitrogen and serum creatinine measurement were done in all patients with BHMS. In addition, kidney ultrasonography was done for evaluation of the renal anomalies and abdominal X-ray was performed for detection of spina bifida and short sacrum. Voiding cystourethrography (VCUG) was used for detection and grading of VUR (12). 

In patients with symptoms of neurologic bladder such as constipation and infrequent voiding, abdominal X-ray was done for assessment of short sacrum and spina bifida. If there were both of them, it was considered as a neurogenic bladder and urodynamic study was done for this group of patients.

In patients with BJHMS and neurogenic bladder who had failure to thrive (FTT), other etiology of FTT was excluded. FTT was diagnosed via calculation of body mass index (BMI) by weight and height adjusted to their age and gender.

## 3. Results

From a total of 58 patients, we found 15 cases with BJHMS, 10 girls and 5 boys. Mean age of patients was 6 ± 2 years. Forty percent of patients with BJHMS had family history of disease, while 83% of subjects with VUR had family history of BJHMS. VUR was concomitant in 60% of patients with BJHMS; whereas 100% of patients with UTI and BJHMS had VUR. Overall, 40% of patients had UTI.

Normal Kidney sonography was seen in 66.7% of all patients. Abnormal findings of sonography were mild (13.3%), moderate (13.3%) and severe hydronephrosis (6.7%). Moreover, this imaging tool revealed 55% abnormality in all patients with VUR. The grade II vesicoureteral reflux was the most common (37.5%) among children with VUR (Table 1). Bilateral reflux was seen in 55.6% of cases. Spina bifida occulta and short sacrum were seen in 46.7% of patients. Neurogenic bladder was also observed in 46.7% of patients with short sacrum. Constipation was frequently occurred in patients with neurogenic bladder (86.7%). BJHMS was associated with FTT in 73% of patients. 

**Table 1.** Patients’ Demographic and Laboratory Characteristic

Variable	Case Number														
	1	2	3	4	5	6	7	8	9	10	11	12	13	14	15
**Age, y**	7	4	7	3	5.5	3.5	10	5	6	8	6.5	3.5	7	4	8
**Gender **	F^a^	F	F	F	F	M^a^	M	M	F	F	M	F	F	M	F
**Complaint **	M.P^a^	A^a^	M.P	A	UTI^a^	M.P	UTI	A	A	UTI	A	dysuria	A	dysuria	UTI
**Family history **	-	-	+	+	-	-	+	-	-	+	-	+	-	-	+
**Treatment duration, mo**	3	1	2	1	1.5	1	4.5	2	2	3	1.5	0	4	2	3
**Enuresis **	-	+	-	-	+	-	+	-	-	+	-	+	-	+	+
**Incontinency**	-	-	-	-	+	-	+	-	-	-	-	+	-	-	+
**Frequency **	-	-	+	-	+	-	+	+	-	-	-	+	-	+	+
**Constipation **	+	+	+	+	+	-	+	+	+	+	+	+	-	+	+
**UTI ** ^**a**^	+	+	-	-	+	-	+	-	-	+	-	+	-	+	+
**Hematuria **	+	+	+	-	+	-	+	-	-	+	-	-	-	-	+
**Brithon score**	6	5	6	7	5	6	7	8	6	5	7	8	7	6	5
**Hydronephrosis (in US^a^)**	mild	-	-	-	moderate	-	severe	-	-	-	-	-	-	mild	moderate
**Vesicouretral reflux grading **	4	2	1	-	2	-	5	-	-	1	-	2	-	3	+
**Vesicouretral reflux laterality**	2	1	1	-	2	-	2	-	-	1	-	2		1	2
**Neurogenic bladder**	+	-	-	-	+	-	+	-	-	+	-	-	+	+	+
**Short sacrum (in KUB^a^)**	-	-	+	-	+	-	+	-	-	+	-	-	+	+	+

^a^Abbreviations: A, arteralgia; F, female; K.U.B, kidney ureter bladder; M, male; MP, Muscle pain; US, ultrasonography; UTI, Urinary tract infection

## 4. Discussion

In the present study, we found a strong association between BJHMS and VUR. Our major novel finding was high prevalence of VUR in children with hypermobility syndrome, 60% of BJHMS had VUR. According to our knowledge, this case series is the first report of association between BJHMS and VUR. Interestingly, 40% of patients with BJHMS had UTI; however, concomitant BJHMS and VUR was 60%. Thus, there were 20% of cases with BJMS and VUR had no history of UTI. It is well known that recurrent UTI associated with VUR; hence, VUR should be considered in cases had BJHMS and UTI. 

Medel et al. demonstrated that collagenous proliferation in primary obstructive megaureter and refluxing megaureter could be related to ureteric smooth muscle cell dysfunction (13). Moreover, Lee et al. showed a greater contribution of type III collagen may play a role in the pathophysiology of refluxing megaureters; it may cause an essentially stiffer ureter and play a role in the lower surgical success in the re-implantation of refluxing megaureters (14). Tokunaka et al. have previously reported the importance of muscle dysplasia to the nonreflux megaureter (15). They revealed that the findings of these dysplastic features of the ureter caused a variety of other congenital disorders of the ureter experienced in their institution (15). When muscle dysplasia was widespread, involving the whole length of the dilated ureter, prevalence of allied renal dimorphism was great as such established either severe renal dysplasia or hypoplasia. Parallel muscle dysplasia was also seen in most of the dome of ureterocele (15).

Two third of our patients had normal urinary tract system in ultrasonography; however, a significant number of cases had VUR. Furthermore, ultrasonography had a low sensitivity value for diagnosis of VUR. The lack of visualization of urethral anomalies reduces the role of ultrasonography in the primary diagnosis of VUR especially in boys. Muensterer et al. reported ultrasound cannot precisely diagnose VUR by morphological changes alone (16). In earlier literature, the accuracy of ultrasonography in comparisons of VCUG has less diagnostic value in detection of VUR, with sensitivities that differ from 26% to 53% and specificities up to 80% (17-19).

BJMHS is more seen in girls than boys (20-22); thus, it seems to be gender-influenced dominant trait disease (23-26). Alike, BJHMS was seen two folds in girls compared to boys in the current case series. Majority of our patients with BJMHS were younger than 7 years old. Some data suggest that BJHMS is more prevalent at earlier age and patients with BJHMS often lead to normal lives without BJHMS or another connective tissue disorder (27).

In our study, genetic had a notable role; family history was seen in many children with BJHMS and VUR, consistent with other studies (28). Although studies for introducing unique gene abnormality have not been successful (20), other connective tissue disorders have been related to some genetic abnormalities (29).

We found a neurogenic bladder more prevalent in BJHMS patients, to our knowledge there is not any evidence on the relation between neurogenic bladder and BJHMS and it is the first time report. However, neurogenic bladder has reported in connective tissue disorder (30). Constipation was seen in many patients with BJHMS and neurogenic bladder. This finding has not been shown in literature, although orthopedic, neurologic and urologic pathology and other problems have been previously reported (14, 24, 31). The seventy percent of patients with BJHMS and neurogenic bladder had FTT and this point may give us attention that it is better to rule out of neurogenic bladder in each patient with BJHMS and FTT.

## 5. Conclusion

Our findings showed an increased frequency of VUR in patients with BJHMS. We suggest that Infants and children with BJHMS should be screened for VUR. However, additional large studies are needed to further examine and confirm the current findings.
